# Health Inequalities in the Diverse World of Self-Employment: A Swedish National Cohort Study

**DOI:** 10.3390/ijerph182312301

**Published:** 2021-11-23

**Authors:** Karl Gauffin, Andrea Dunlavy

**Affiliations:** Department of Public Health Sciences, Stockholm University, 106 91 Stockholm, Sweden; andrea.dunlavy@su.se

**Keywords:** self-employment, health inequalities, Sweden

## Abstract

With labor being a central social determinant of health, there is an increasing need to investigate health inequalities within the heterogenous and growing population in self-employment. This study aimed to longitudinally investigate the relationship between income level, self-employment status and multiple work-related health indicators in a Swedish national cohort (*n* = 3,530,309). The study investigated the relationship between self-employment status and health outcomes later in life. All poor health outcomes, with the exception of alcohol-related disorders, were more common in the self-employed population, compared to the group in regular employment. The income gradient, however, was more pronounced in the group with regular employment than the groups in self-employment. The study found clear connections between low income and poor health in all employment groups, but the gradient was more pronounced in the group in regular employment. This suggests that income has a weaker connection to other types of health promoting resources in the self-employed population. Potentially, lacking social and public support could make it difficult for unhealthy individuals to maintain low-income self-employment over a longer time period.

## 1. Introduction

Studies on the interconnections between labor market development and public health are situated in a highly volatile research field. Employment and working conditions are central social determinants of health, not only because they provide the worker with an income, but also because the work place constitutes an important arena for health promoting or health damaging job tasks, activities and social relationships. In the European welfare state, wage labor has become a major source of social security, in which the worker, the employer and the state share the cost for social insurances that provide the worker with economic compensation in times of sickness, parental leave, and retirement. As many countries now report an increase in non-standard employment arrangements [1], this should also be of concern from a public health perspective, as these type of work arrangements have been shown to be potentially adverse for population health [2,3,4]. Along with the other Nordic countries, Sweden is an interesting case in point, as it has earned international recognition for its universal and relatively egalitarian welfare state, which should make the country well-equipped to counteract the global trends of increasing economic and social inequalities. However, since the 1990s, a number of political reforms, including far-reaching privatization and deregulation measures in the Swedish welfare system and the labor market, have coincided with a significant increase in income and health-related inequalities [5,6].

A central element of the political and institutional processes that challenge standard employment arrangements as the norm of Western labor markets is the decoupling of workers from their employers [7]. The employer becomes an outsourcer with limited or non-existent responsibility for social security and income protection, while workers may be required to reconstruct themselves as self-employed entrepreneurs within an increasingly insecure labor market [8]. Self-employed workers also may not benefit from occupational health and safety legislation to the same extent as workers in regular employment. This implies a shift in responsibility, where the worker bears the risks associated with work instead of the employer [9]. As such, a low-paid worker in self-employment may be in a particularly vulnerable position, as they often operate outside the coverage of any social safety net, which in the Nordic countries usually apply to all workers in standard employment, regardless of their income level. As precarious self-employment co-exists with the more privileged segments of self-employed workers, it remains important to pay close attention to income levels, as well as job security, job quality and other arrangements, when investigating the meaning of self-employment for the individual worker [7,10].

Given the complexity of precarious employment and its connection to self-employment, it is unsurprising that its effects on health are also multifaceted. Previous studies have demonstrated a connection between precarious employment and general and cause-specific poor health [4,11,12]. Studies on cause-specific morbidity may consider the different dimensions of precarious employment and their meaning for health. For example, a sub-standard work environment may increase the risk for disorders of the musculoskeletal system [13], while the subjective experiences of stress, insecurity, powerlessness and unfairness are likely to have a negative impact on mental health [3,14,15,16], and alcohol consumption [17]. Self-employment also has connections to health, as demonstrated by a number of studies [18,19,20]. Toivanen and colleagues [21] investigated the relationship between self-employment and mortality in Sweden and demonstrated that compared to workers in standard employment, mortality rates among the self-employed depend on industry sector and type of self-employment. While those with a limited liability company had lower mortality compared to standard employees, mortality was higher in sole proprietors in some occupational sectors, such as trade, transportation and welfare services. These results demonstrate heterogeneity among the self-employed and underline the importance of considering additional variables when examining relationships with health. Efforts to investigate the relationships between self-employment, precariousness and health have highlighted the particular importance of longitudinal study designs [22]. Not only are the mid- or long-term effects of precarious employment on health difficult to capture in a cross-sectional study, but information on the duration of precariousness will also be of great significance for any study conclusion. Short-term experiences of precarious employment arrangements, perhaps in the beginning of the professional career, will likely not have the same meaning as longstanding precarious attachment to the labor market.

The present study contributes to this limited body of research by investigating how different types of self-employment trajectories and levels of earned income are related to all-cause and cause-specific inpatient care and mortality in a Swedish register-based birth cohort. With the exception of inpatient care for alcohol-related disorders, all poor health outcomes were more commonly observed in the self-employed compared to those in regular employment. An income gradient in the risk for poor health was observed among the self-employed and those in regular employment; however, the gradient was most pronounced among those in regular employment, suggesting that other work-related health determinants, such as job control or satisfaction, may play a more central role in influencing health among the self-employed.

## 2. Materials and Methods

Swedish population registers include a large number of social, educational and health-related indicators and provide researchers with excellent data for longitudinal population-based studies. Every Swedish resident is given a unique personal identification number (PIN) at the time of birth or immigration. The PINs enable record linking, which makes it possible to follow a person from birth to death in the population registers. For privacy reasons, the PINs are anonymized when used in research. The study has been approved by the regional ethics committee in the Stockholm region (project number 2018/1274-31).

The study population includes all men and women who were born between 1941 and 1970 and registered as residents of Sweden between 1996 and 2003 (*n* = 3,530,309). All demographic data, including gender and country of birth, were retrieved from the Medical Birth Register and the Longitudinal Integration Database for Health Insurance and Labor Market Studies (LISA).

Employment status was measured for eight consecutive years from 1996 to 2003. In the beginning of the employment follow-up, the study population was aged 26–55 years, and in the end, they were aged 33–62 years. The members of the study population were classified according to their modal employment status during the eight-year follow-up period.

This classification resulted in four groups: Regular employment, self-employment in sole proprietorship, self-employment as owner of a limited liability company and other or no information (e.g., people in unemployment, early retirement or students). In addition, in order to assess the employment type transitions of the self-employed, this population was further categorized according to their employment history before becoming self-employed, which resulted in four additional groups: Those in stable self-employment (i.e., self-employed for all eight years of follow-up), those transferring into self-employment from unemployment, those transferring into self-employment from regular employment, and other. The last category included the population with self-employment as the modal status of employment, but who transferred from self-employment to either regular employment or unemployment. Finally, the population was divided into income quintiles according to average earned income in the eight years of follow-up. All information on employment status and income was retrieved from the LISA database.

Information on mortality, all-cause inpatient care and cause-specific inpatient care was retrieved from the cause of death register and the patient register during the years 2004 to 2016. All-cause inpatient care excluded diagnostic codes for uncomplicated child deliveries (ICD-10: O80-O82). In the beginning of the health follow-up, the study population was aged 24 to 62 years and in the end, they were aged 46 to 75 years. Cause-specific inpatient care referred to inpatient care due to (i) diseases of the circulatory system (ICD-10: I00-I99), (ii) diseases of the musculoskeletal system (ICD-10: M00-M99) and alcohol-related disorders (ICD-10: F100-F109, X45, Y15).

Incidence rates per 1000 person-years were calculated for all health outcomes. Excluding the population with other or no employment information, crude incidence rate ratios were provided to compare the population in regular employment with the population in sole proprietorship and limited liability company owners. Cox regression models were used to calculate hazard ratios (HR) with 95% confidence intervals (CI) for the health outcomes by two sets of variables: (i) income quintile and employment status and (ii) employment history and type of self-employment. Entry date was defined as first day of health follow-up (1 January 2004) and the study population was followed until death, first instance of inpatient care or end of follow-up (31 December 2016). All regression analyses were adjusted for year of birth and gender. In the analysis of income and employment status, all models included a multiplicative interaction term for employment type and income quintile, and hazard ratios were calculated using linear combinations of coefficients to compare differences in the relative risks for mortality and inpatient care between groups with different employment types and income levels. The population in regular employment in the highest income quintile was used as the common reference group. The analysis of employment history and type of self-employment was designed in a similar way, but using an additional population category—those with eight years of stable regular employment—as the reference category. All statistical analysis was performed in Stata v15.

## 3. Results

Table 1 shows the demographic profile of the total population and the population in self-employment. The self-employed population represents around 7 percent of the total population and includes higher proportions of men, low-educated, natives and lower income quintile groups compared to the total population. Around two thirds of the self-employed population were in sole proprietorship and one third were owners of limited liability companies. Almost half of the self-employed population had been in self-employment throughout the eight years of employment follow-up, whereas about 10 percent transferred into self-employment from unemployment and about 27 percent from regular employment.

Table 2 shows the same demographic profiles for the two groups in self-employment. Female gender, lower education, non-native background, lower earned income and a history of unemployment were more common in the group in sole proprietorship compared to owners of limited liability companies.

Table 3 shows the total number of cases and crude incidence rates per 1000 person-years and incidence rate ratios by employment status for death and first instance of all-cause inpatient care and three types of cause-specific inpatient care. With the exception of inpatient care for alcohol-related disorders, all poor health outcomes were more common in the self-employed groups compared to the population in regular employment. Comparing the self-employed groups with each other, the incidence rates were comparable for all health outcomes except inpatient care related to musculoskeletal disorders, which was more common among limited liability company owners compared to the group in sole proprietorship.

**Figure 1:**
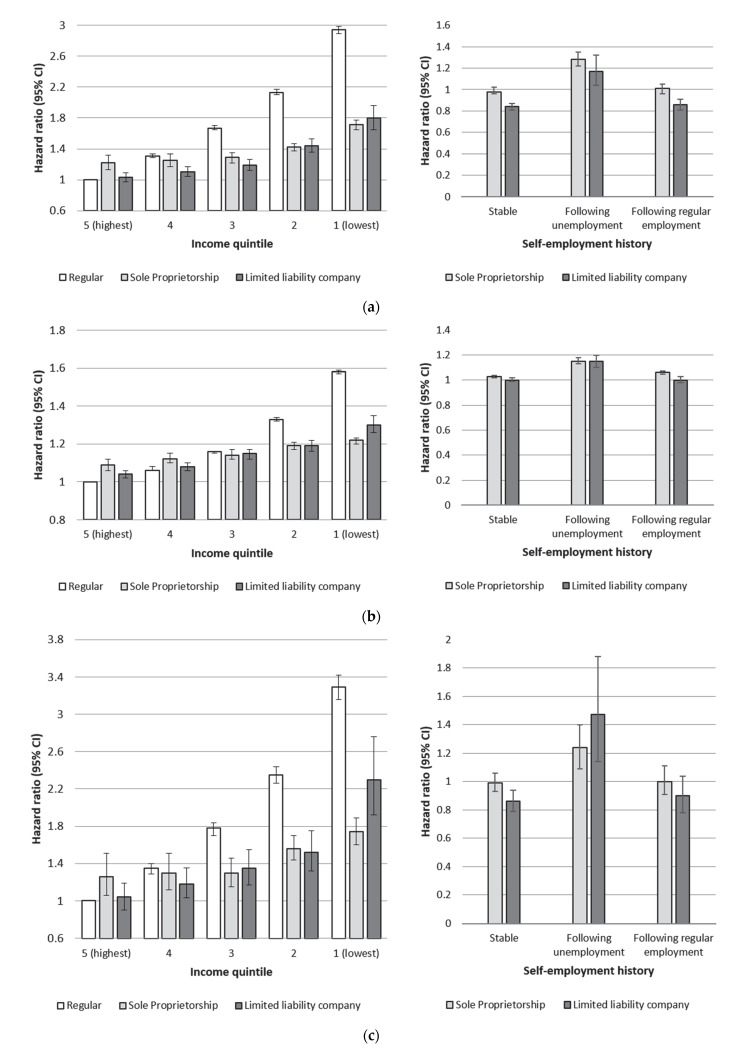

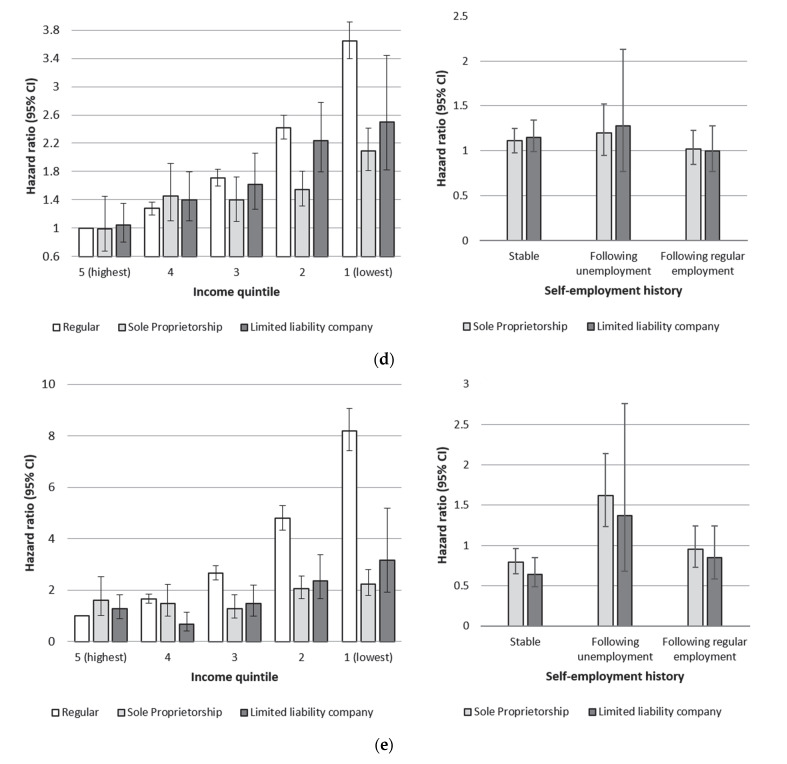
(**a**) Hazard ratios (95% CI) for all-cause mortality by income quintile and type of self-employment. (**b**) Hazard ratios (95% CI) for all-cause inpatient care by income and type of self-employment. (**c**) Hazard ratios (95% CI) for inpatient care related to diseases of the circulatory system by income quintile and type of self-employment. (**d**) Hazard ratios (95% CI) for inpatient care related to diseases of the musculoskeletal system by income quintile and type of self-employment. (**e**) Hazard ratios (95% CI) for inpatient care related to alcohol-related disorders by income quintile and type of self-employment.

Figure 1a–e show hazard ratios related to employment status, income quintile and employment history for all five health outcomes, adjusted for year of birth and gender (corresponding tables are provided as supplementary materials). All analyses of income and employment status use the population in regular employment and the highest income quintile as the reference group, whereas the analyses of employment history and type of self-employment use the population in stable regular employment as the reference category.

To varying degrees, a clear income gradient was evident for all five health outcomes. The excess risk in the lowest income quintile in regular employment compared to the highest income quintile reached HR 1.58 (95% CI 1.57–1.59) for total inpatient care and HR 8.19 (95% CI 7.43–9.05) for inpatient care for alcohol-related disorders. The income gradient was also present in the self-employed groups, but it was not as pronounced as that observed in the population with regular employment. Comparing the two self-employed groups with each other, the income gradient in risks for mortality and all-cause inpatient care were similar. In terms of cause-specific inpatient care, a slightly higher risk of inpatient care for circulatory diseases was observed among owners of limited liability companies with low income compared to the same income group among sole proprietors (HR = 2.30; 95% CI: 1.92–2.76 and HR = 1.74; 95% CI: 1.60–1.89, respectively). Post-estimation tests confirmed that the risk among limited liability company owners in the lowest income quintile was significantly higher than the observed risk among sole proprietors in the lowest income quintile (*p* < 0.01). Comparing the highest income quintiles, however, showed that the group in sole proprietorship had a slightly higher risk of mortality (HR = 1.22, 95% CI: 1.1.3–1.32), inpatient care for circulatory disease (HR = 1.26, 95% CI: 1.06–1.51) and alcohol-related disorders (HR = 1.60, 95% CI: 1.01–2.52) compared to the reference group in regular employment, whereas the limited liability company group in the highest income quintile showed no elevated risk. Both the highest income sole proprietors and limited liability company owners showed a marginally elevated risk for all-cause inpatient care relative to the highest income group in regular employment. Stratified analyses for men and women were conducted separately in order to control for any potential effect modification related to gender. The income gradients in all employment groups were slightly more pronounced among men compared to women, but that was the only gender-related difference (gender-stratified analyses provided as supplementary materials).

The analysis of employment history found a slightly increased risk of mortality in the groups transferring to self-employment from unemployment (HR = 1.28, 95% CI: 1.22–1.35 and HR = 1.17, 95% CI: 1.04–1.32 for those in sole proprietorship and owners of limited liability companies, respectively). Among limited liability company owners, the risk was slightly lower both among those transferring from regular employment (HR = 0.86, 95% CI: 0.81–0.91) and those in stable self-employment (HR = 0.84, 95% CI: 0.81–0.87). Apart from a slight tendency for higher risks among sole proprietors and those transferring into self-employment from unemployment, there were no large risk differences for inpatient care related to type of self-employment and employment history (see Figure 1b–e).

## 4. Discussion

This Swedish cohort study, including 3.5 million working-age individuals, has made use of national register data to shed light on the relationships between earned income level, self-employment, employment history and five different health outcomes. Compared to the population in regular employment, the self-employed population was found to have higher crude mortality rates and higher crude incidence rates for the first instance of all-cause inpatient care and inpatient care related to diseases of the circulatory system and musculoskeletal system. However, regression analyses, adjusted for gender and year of birth, did not find evidence of a more vulnerable health situation for low-income workers in self-employment, as the highest risks for mortality and all types of inpatient care were found in the lowest income quintile in regular employment. In addition, the self-employed group with a history of unemployment had a slightly larger risk for mortality and different types of inpatient care.

The identified income gradient observed across all employment groups is in line with decades of previous research finding a stepwise relationship between income and health. The income gradient with regard to alcohol-related inpatient care was the most pronounced across all employment types, which could possibly be explained by the likelihood that the type of severe alcohol misuse that precedes alcohol-related inpatient care may have a negative impact on income. The general risk of reversed causation between poor health and income was minimized by allowing the employment follow-up to precede the health follow-up, but in the specific case of alcohol and other types of substance misuse, years of problems prior to hospitalization could be a distinct possibility.

The weaker income gradients observed in the self-employed groups suggest that, compared to workers in regular employment, income may have less significance for health among the self-employed. This could be an indication of income having a weaker connection to other types of health promoting resources in self-employed groups, such as job control and flexibility over work tasks, which are important factors that influence work-related health. Prior studies have shown that self-employed persons report greater job satisfaction and job control than do paid employees [21,23], but may also face greater work demands and less social support [24]. Although findings remain inconclusive in terms of selection effects into self-employment [18], there is some evidence for better health outcomes among the self-employed [21,24,25], which lends support to the observed weaker income gradient among the self-employed groups in the current study. In addition, the long employment follow-up of eight consecutive years could itself introduce a selection effect, as it might be less likely that an unhealthy person would be able to keep a low-income business running in combination with the relative insecurity that sole proprietorship may entail. By contrast, regular employment provides the employee—even if low-income and unhealthy—with some security, which may permit employees to remain in regular employment for longer durations. Examination of the employment history prior to entering into self-employment adds additional insights, as the group in stable self-employment, who arguably may have the best health among the self-employed given the stability of their employment over time, had equal or lower risk for mortality and most types of inpatient care compared to the population in regular employment. In line with previous studies [26,27], we similarly observed a weaker income gradient among women compared to men. This finding may be due to the fact that income from work is a better indicator of material resources for men than for women, as men generally still contribute more to household consumption ability. Studies have also suggested that psychosocial and social status factors may be more important health determinants for women than for men [27].

Although the income gradient in health was most pronounced among persons in standard employment, comparison of the highest income groups across employment types showed slightly elevated risks for mortality, all-cause inpatient care, and inpatient care for circulatory diseases and alcohol-related disorders among sole proprietors, whereas no excess risk was found among limited liability company owners relative to high-income earners in regular employment. This elevated risk in high-income sole proprietors could be another indication of a weaker relationship between income and health in this group, but in this instance to the disadvantage of sole proprietors. Furthermore, the top income group of sole proprietors may represent a particular group of self-employed individuals that, in spite of their high income, face working conditions that may be detrimental to health.

The self-employed are a heterogeneous group, with great variation in working conditions and demands, as well as professional qualifications, which makes it difficult to generalize findings. Studies with more detailed data and greater capacity to study this individual unobserved heterogeneity in the self-employed population will add important contributions to the research field. Another important research area will be the investigation of self-employment in the time following the global COVID-19 pandemic. Ongoing research during the pandemic has been reporting particular vulnerabilities among the self-employed, including limited eligibility for state income support [28,29].

This study utilized longitudinal population-based data, which provided high-quality information on employment type and earned income over an extended follow up period, and enabled a more robust assessment of employment type. In addition, to the authors’ knowledge this is the first study to explicitly examine the relationship between self-employment and health across income quintile groups.

Despite the strengths of the register data utilized, we were not able to directly assess for working conditions or quality, which may play a large role in experiences of job stress and thus have strong implications for health. In addition, we cannot rule out the possibility of health selection into self-employment. Finally, in spite of the many strengths of national register data, it is less well equipped to capture more precarious forms of self-employment for individuals with zero-hour contracts or who are members of the gig economy. In addition, short-term labor immigrants who are not registered as residents in the country are not included in the population registers.

## 5. Conclusions

Our findings confirm previous research that shows an income gradient in health among employed and self-employed workers. Despite a weaker gradient observed among the self-employed, it remains vital to investigate relationships between health and employment status, which include the self-employed, particularly as this group is likely to comprise increasingly larger proportions of the labor market in the near future.

## Figures and Tables

**Table 1:** Description of population.

	Total Population		Population in Self-Employment	
	***n***	**%**		
**Total (*n*)**	3,530,309		245,807	
**Sex**				
Men	1,788,578	50.7%	173,344	70.5%
Women	1,741,731	49.3%	72,463	29.5%
**Education**				
Post-secondary	1,443,401	40.9%	87,094	35.4%
No post-secondary	2,046,216	58.0%	156,417	63.6%
**Country of origin**				
Natives	3,070,133	87.0%	217,276	88.4%
Nordic countries	147,060	4.2%	7139	2.9%
Europe and other high-income countries	194,079	5.5%	12,246	5.0%
Other	119,037	3.4%	9146	3.7%
**Income quintile**				
5 (highest)	706,052	20.0%	33,567	13.7%
4	705,990	20.0%	39,103	15.9%
3	706,135	20.0%	45,953	18.7%
2	706,010	20.0%	71,617	29.1%
1 (lowest)	706,122	20.0%	55,567	22.6%
**Type of self-employment**				
Sole proprietorship	-	-	161,802	65.8%
Limited liability company owner	-	-	84,005	34.2%
**Employment history before self-employment**				
Stable self-employment	-	-	119,205	48.5%
Unemployment followed by self-employment	-	-	24,198	9.8%
Regular employment followed by self-employment	-	-	66,878	27.2%
Other ^a^	-	-	35,526	14.5%

^a^ Self-employment is the modal employment status, but latest transition was to unemployment or regular employment.

**Table 2:** Self-employment in population.

	Sole Proprietorship		Limited Liability Company	
	*n*	%		
**Total (*n*)**	161,802		84,005	
**Sex**				
Men	111,980	69.2%	61,364	73.0%
Women	49,822	30.8%	22,641	27.0%
**Education**				
Post-secondary	54,693	33.8%	32,401	38.6%
No post-secondary	105,144	65.0%	51,273	61.0%
**Country of origin**				
Natives	138,616	85.7%	78,660	93.6%
Nordic countries	4979	3.1%	2160	2.6%
Europe and other high-income countries	9938	6.1%	2308	2.7%
Other	8269	5.1%	877	1.0%
**Income quintile**				
5 (highest)	11,740	7.3%	21,827	26.0%
4	17,418	10.8%	21,685	25.8%
3	27,625	17.1%	18,328	21.8%
2	55,456	34.3%	16,161	19.2%
1 (lowest)	49,563	30.6%	6004	7.1%
**Employment history before self-employment**				
Stable self-employment	75,370	46.6%	43,835	52.2%
Unemployment followed by self-employment	20,568	12.7%	3630	4.3%
Regular employment followed by self-employment	45,401	28.1%	21,477	25.6%
Other ^a^	20,463	12.7%	15,063	17.9%

^a^ Self-employment is the modal employment status, but latest transition was to unemployment or regular employment.

**Table 3:** Mortality rate and incidence of first event of inpatient care by history of self-employment.

	Person-Years	Total Number of Cases	Incidence Rate per 1000 Person Years (95% CI)	Incidence Rate Ratio (95% CI)
	161,802		84,005	
**Mortality**				
Regular employment	40,219,528	158,591	3.9 (3.9–4.0)	1
Sole proprietorship	2,205,638	10,240	4.6 (4.6–4.7)	1.18 (1.15–1.20)
Limited liability company owner	1,146,283	5184	4.5 (4.4–4.7)	1.15 (1.11–1.18)
**All-cause inpatient care**				
Regular employment	28,044,631	1,418,275	50.5 (50.4–50.6)	1
Sole proprietorship	1,505,501	82,632	54.5 (54.0–54.8)	1.09 (1.08–1.09)
Limited liability company owner	789,953	43,252	54.8 (54.2–55.2)	1.10 (1.09–1.11)
**Inpatient care related to diseases of the circulatory system**				
Regular employment	35,002,244	27,196	0.78 (0.77–0.79)	1
Sole proprietorship	1,884,634	1863	0.99 (0.94–1.03)	1.27 (1.21–1.33)
Limited liability company owner	969,622	978	1.00 (0.95–1.07)	1.30 (1.21–1.38)
**Inpatient care related to musculoskeletal system**				
Regular employment	35,436,759	8428	0.24 (0.23–0.24)	1
Sole proprietorship	1,931,769	558	0.29 (0.27–0.31)	1.21 (1.11–1.32)
Limited liability company owner	1,000,703	320	0.32 (0.28–0.36)	1.34 (1.20–1.50)
**Inpatient care related to alcohol-related disorders**				
Regular employment	37,210,269	4789	0.13 (0.13–0.13)	1
Sole proprietorship	2,040,716	266	0.13 (0.12–0.15)	1.01 (0.89–1.14)
Limited liability company owner	1,061,683	121	0.11 (0.09–0.14)	0.89 (0.73–1.06)

## Data Availability

As the data consist of sensitive personal information, the data cannot be made publicly available. Any data-related inquiries can be emailed to the corresponding author.

## Supplementary Materials

The following are available online at https://www.mdpi.com/article/10.3390/ijerph182312301/s1, Table S1: Hazard ratios (95% CI) for all-cause mortality by income quintile and type of self-employment^1^, Table S2. Hazard ratios (95% CI) for all-cause inpatient care by income and type of self-employment, Table S3. Hazard ratios (95% CI) for inpatient care related to diseases of the circulatory system by income quintile and type of self-employment, Table S4. Hazard ratios (95% CI) for inpatient care related to diseases of the musculoskeletal system by income quintile and type of self-employment, Table S5. Hazard ratios (95% CI) for inpatient care related to alcohol related disorders by income quintile and type of self-employment.
