# Supplementary material for: Health Inequalities in the Diverse World of Self-Employment: A Swedish National Cohort Study
Source: Int J Environ Res Public Health. 2021 Nov 23;18(23):12301. doi: 10.3390/ijerph182312301 (PMC8656803; doi:10.3390/ijerph182312301)
Supplement: Supplementary file 1 [file ijerph-18-12301-s001.zip › ijerph-1434411 Supplementary Materials.pdf]

International Journal of
Environmental Research and Public Health

MDPI

Supplementary material

Table S1. Hazard ratios (95% CI) for all-cause mortality by income quintile and type of self-employment[1]

	Regular employment		Sole proprietorship		Limited liability company	
	**HR**	**95% CI**	**HR**	**95% CI**	**HR**	**95% CI**
**Income quintile**						
5 (highest)	1	ref	1.22	1.13-1.32	1.03	0.97-1.09
4	1.31	1.29-1.33	1.25	1.17-1.33	1.10	1.04-1.17
3	1.67	1.65-170	1.29	1.22-1.35	1.19	1.12-1.26
2	2.13	2.10-2.17	1.42	1.37-1.47	1.44	1.36-1.53
1 (lowest)	2.94	2.89-2.99	1.71	1.65-1.77	1.80	1.65-1.96
**Employment history**						
Stable regular employment	1	ref	-	-	-	-
Stable self-employment			0.98	0.96-1.02	0.84	0.81-0.87
Self-employment following:						
unemployment			1.28	1.22-1.35	1.17	1.04-1.32
regular employment			1.01	0.96-1.05	0.86	0.81-0.91

[1] Tables S1-S5 are adjusted for year of birth and gender

Table S2. Hazard ratios (95% CI) for all-cause inpatient care by income and type of self-employment

	Regular employment		Sole proprietorship		Limited liability company	
	**HR**	**95% CI**	**HR**	**95% CI**	**HR**	**95% CI**
**Income quintile**						
5 (highest)	1	ref	1.09	1.06-1.12	1.04	1.02-1.06
4	1.06	1.06-1.08	1.12	1.10-1.15	1.08	1.06-1.10
3	1.16	1.15-1.16	1.14	1.12-1.17	1.15	1.12-1.17
2	1.33	1.32-1.34	1.19	1.17-1.21	1.19	1.16-1.22
1 (lowest)	1.58	1.57-1.59	1.22	1.20-1.23	1.30	1.26-1.35
**Employment history**						
Stable regular employment	1	ref	-	-	-	-
Stable self-employment	-	-	1.03	1.02-1.04	1.00	0.99-1.02
Self-employment following:						
unemployment	-	-	1.15	1.13-1.18	1.15	1.10-1.20
regular employment	-	-	1.06	1.05 -1.07	1.00	0.98-1.03

Table S3. Hazard ratios (95% CI) for inpatient care related to diseases of the circulatory system by income quintile and type of self-employment

	Regular employment		Sole proprietorship		Limited liability company	
	**HR**	**95% CI**	**HR**	**95% CI**	**HR**	**95% CI**
**Income quintile**						
5 (highest)	1	ref	1.26	1.06-1.51	1.04	0.90-1.19
4	1.35	1.29-1.40	1.30	1.12-1.51	1.18	1.03-1.35
3	1.78	1.70-1.84	1.30	1.15-1.46	1.35	1.17-1.55
2	2.35	2.26-2.44	1.56	1.44-1.70	1.52	1.32-1.75
1 (lowest)	3.29	3.16-3.42	1.74	1.60-1.89	2.30	1.92-2.76
**Employment history**						
Stable regular employment	1	ref	-	-	-	-
Stable self-employment	-	-	0.99	0.93-1.06	0.86	0.79-0.94
Self-employment following:						
unemployment	-	-	1.24	1.09-1.40	1.47	1.14-1.88
regular employment	-	-	1.00	0.91-1.11	0.90	0.78-1.04

Table S4. Hazard ratios (95% CI) for inpatient care related to diseases of the musculoskeletal system by income quintile and type of self-employment

	Regular employment		Sole proprietorship		Limited liability company	
	**HR**	**95% CI**	**HR**	**95% CI**	**HR**	**95% CI**
**Income quintile**						
5 (highest)	1	ref	0.99	0.67-1.45	1.04	0.80-1.35
4	1.28	1.18-1.37	1.45	1.10-1.91	1.40	1.10-1.79
3	1.71	1.59-1.83	1.40	1.09-1.72	1.62	1.27-2.06
2	2.42	2.26-2.60	1.54	1.31-1.80	2.24	1.79-2.78
1 (lowest)	3.65	3.40-3.92	2.09	1.81-2.41	2.50	1.82-3.44
**Employment history**						
Stable regular employment	1	ref	-	-	-	-
Stable self-employment	-	-	1.11	0.98-1.25	1.15	0.99-1.34
Self-employment following:						
unemployment	-	-	1.20	0.95-1.52	1.28	0.77-2.13
regular employment	-	-	1.02	0.85-1.23	1.00	0.77-1.28

Table S5. Hazard ratios (95% CI) for inpatient care related to alcohol related disorders by income quintile and type of self-employment

	Regular employment		Sole proprietorship		Limited liability company	
	**HR**	**95% CI**	**HR**	**95% CI**	**HR**	**95% CI**
**Income quintile**						
5 (highest)	1	ref	1.60	1.01-2.52	1.27	0.88-1.83
4	1.66	1.49-1.84	1.48	0.99-2.21	0.68	0.41-1.14
3	2.67	2.41-2.96	1.29	0.91-1.82	1.49	1.00-2.20
2	4.79	4.34-5.28	2.05	1.66-2.54	2.37	1.67-3.37
1 (lowest)	8.19	7.43-9.05	2.24	1.79-2.79	3.17	1.93-5.20
**Employment history**						
Stable regular employment	1	ref	-	-	-	-
Stable self-employment	-	-	0.79	0.65-0.96	0.64	0.49-0.85
Self-employment following:						
unemployment	-	-	1.62	1.23-2.14	1.37	0.68-2.76
regular employment	-	-	0.95	0.73-1.24	0.85	0.58-1.24
